# A New Neurological Screening Approach for Diagnosing Brainstem Infarction Using the Calling Method and Familiar Voices

**DOI:** 10.3390/medicina59071344

**Published:** 2023-07-21

**Authors:** Yuzuru Ohshiro

**Affiliations:** Department of Internal Medcine, Omoromachi Medical Center, Naha City 900-0011, Okinawa, Japan; ooshiro@aol.com

**Keywords:** brainstem infarction, dysarthria, stroke screenings, case report

## Abstract

This report proposes a new approach to assess dysarthria in patients with brainstem infarction by involving familiar individuals. Collaboration provides valuable insights compared to subjective traditional methods. A man in his 70s presented with resolved positional vertigo. Standard neurological tests showed no abnormalities, and inquiries with the patient’s friend did not reveal voice changes. While inquiring about voice changes with family, friends, and acquaintances is a common practice in clinical settings, our approach involved the patient calling out to his friend from a distance. Despite the physician detecting no abnormalities, the friend noticed a lower voice. Subsequent magnetic resonance imaging (MRI) confirmed brainstem infarction. Early and subtle symptoms of brainstem infarction pose a detection challenge and can lead to serious outcomes if overlooked. This report provides the first evidence that distance calling can detect subtle voice changes associated with brainstem infarction potentially overlooked by conventional neurological examinations, including inquiries with individuals familiar with the patient’s voice. Detecting brainstem infarction in emergency department cases is often missed, but conducting MRIs on every patient is not feasible. This simple method may identify patients overlooked by conventional screening who should undergo neuroimaging such as MRI. Further research is needed, and involving non-professionals in assessments could significantly advance the diagnostic process.

## 1. Introduction

Brain infarction, also known as ischemic stroke, is a significant healthcare concern due to its high prevalence and substantial economic impact. In the United States, approximately 800,000 individuals annually experience either a new or recurrent stroke [1,2]. Diagnostic errors in stroke management contribute to adverse outcomes, and the National Academy of Medicine has highlighted the likelihood of diagnostic errors occurring in the lives of most Americans, with approximately 50% of these errors leading to serious disability or death. Failure to promptly diagnose and treat ischemic stroke can lead to higher rates of morbidity and mortality, especially when acute thrombolytic or endovascular therapy is not administered within the time-sensitive treatment windows [1,3,4].

Brainstem infarcts constitute 10% of all ischemic brain strokes and encompass a range of syndromes that are challenging to diagnose and affect the midbrain, pons, and medulla oblongata. They can manifest with a wide array of symptoms, including cranial nerve impairments (III to XII), respiratory and cardiac dysfunction, decreased consciousness, and even death [5,6,7]. Early diagnosis is crucial due to the high mortality and morbidity associated with brainstem infarction [8,9]. Brainstem infarction impacts the functionality of the muscles responsible for mouth, tongue, and throat movements. Thoroughly assessing speech motor deficits stands as a pivotal screening approach for the early detection of brainstem infarction. Several speech tests have been reported as effective tools for identifying these deficits associated with stroke. These assessments typically involve analyzing speech patterns during natural conversations or instructing the patients to articulate specific words or phrases to evaluate their pronunciation skills [10,11,12]. While these evaluation methods may include soliciting observations from the patient’s family members regarding any deviations from the patient’s typical speech, the primary responsibility for identifying speech motor deficits lies with the attending physician. Importantly, the manifestations resulting from brainstem infarction can sometimes be very subtle, with no clear deficits apparent during neurological examination [6,9]. In this context, this case report introduces a novel approach to assess dysarthria in patients with brainstem infarction, focusing on collaboration with individuals familiar with the patient. Traditional methods rely on professional assessments, including inquiries with individuals familiar with the patient’s voice [10,11,12]. However, these methods may overlook subtle voice changes associated with early brainstem infarction, leading to potential diagnostic challenges and adverse outcomes. Detecting brain stroke in emergency department cases is often missed, but conducting magnetic resonance imaging (MRI) on every patient is not feasible.

To address this issue, an approach involving non-professionals in the assessment process is proposed, specifically using the patient’s calling out to individuals who are familiar with the patient’s normal voice as an indicator of voice changes. This approach aims to identify patients who may have been overlooked by conventional screening methods and require further neuroimaging, such as MRI. By leveraging the observations of non-professionals, we can enhance the diagnostic accuracy for identifying dysarthria associated with brainstem infarction and provide timely and appropriate interventions for improved patient outcomes.

## 2. Case Presentation

**Figure 1:**
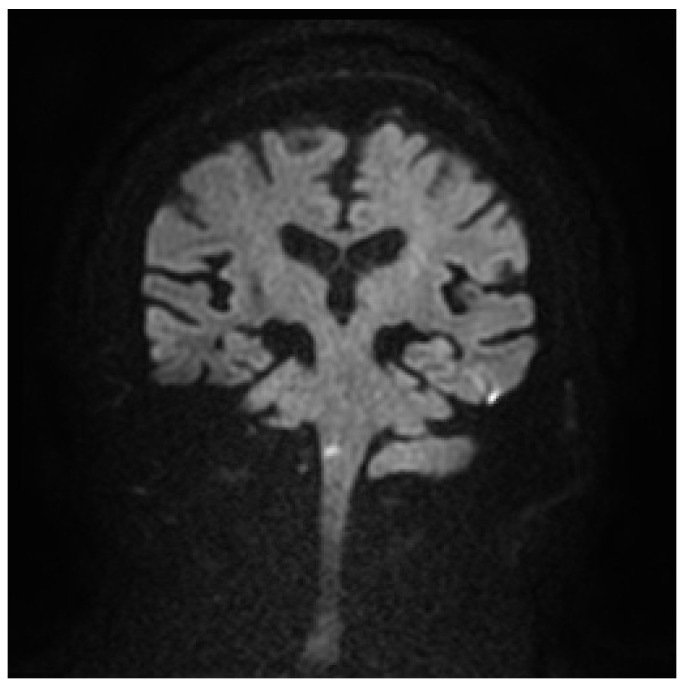
Diffusion-weighted magnetic resonance imaging showed evidence of acute brainstem infarction in the right lateral medulla.

A man in his 70s presented to the emergency room with positional vertigo upon waking up. Six hours later, when he arrived at the hospital, the vertigo had resolved, and there were no apparent sensory deficits or motor paralysis. The head impulse test, which assesses the vestibulo-ocular reflex, was performed and yielded negative results, indicating no abnormal eye movements. Standard assessments for speech disorders, including tests for explosive sounds and consonant repetitions, did not indicate any abnormalities. Examination of the pharynx showed no signs of the “curtain sign”, which is associated with certain neurological conditions such as brainstem stroke. The patient did not have dementia and was able to follow instructions accurately. These findings, along with the absence of other focal neurological deficits, suggested no immediate evidence of a stroke or significant neurological impairment. During the examination, the patient was accompanied by his friend, who stated that his voice sounded normal without any changes. Since the friend mentioned that the patient occasionally calls out to him from a nearby room, the patient was asked to call out to his friend from a distance of approximately 5 m. The examining physician did not detect any abnormalities in the patient’s voice. However, the friend clearly noticed that the patient’s voice was lower than usual. A subsequent head MRI revealed an acute brainstem infarction in the lateral medulla (Figure 1). After three hours, the patient’s voice returned to normal.

## 3. The “Calling Method” and Results

A “Calling Method” was employed as a supplementary assessment to further evaluate the patient’s voice. During the conventional neurological evaluation, both the physician and the friend did not detect any apparent abnormalities in the patient’s voice. However, recognizing the potential limitations of the conventional evaluation, the “Calling Method” was utilized.

The patient was instructed to call out to an observer positioned 5 m away. The observer, who had familiarity with the patient’s usual voice characteristics from a distance, carefully listened for any changes in the patient’s voice during the call-out. Meanwhile, the examining physician stood approximately 1 m away, observing the patient’s facial expressions and voice during the call-out. During the “Calling Method” assessment, the physician did not perceive any abnormalities in the patient’s voice, as the patient’s facial expression appeared normal, and each word spoken during the call-out was clear. However, the friend, who was more attuned to the patient’s usual voice characteristics, specifically noted that the patient’s voice was lower than usual and possibly slightly hoarse, though the degree of hoarseness was challenging to determine precisely.

To validate these observations, a reevaluation of the “Calling Method” was conducted three hours later. During this reevaluation, the patient’s voice was noticeably higher compared to the initial examination, which the physician clearly detected. Furthermore, upon reevaluation, the physician also noticed that the patient’s voice during the initial examination had some degree of hoarseness compared to the second voice assessment. The friend confirmed that the patient’s voice had returned to its usual state.

## 4. Discussion

The consequences of delayed diagnosis in patients presenting with subtle stroke symptoms are significant. Failure to recognize a stroke can result in the exclusion of time-sensitive treatments, such as thrombolytic therapy for acute ischemic stroke. Patients with delayed stroke diagnosis also tend to have less favorable outcomes at one year compared to those accurately diagnosed [4,13]. This report provides the first evidence that distance calling can detect subtle voice changes associated with brainstem infarction potentially overlooked by conventional neurological examinations, including inquiries with individuals familiar with the patient’s voice. This simple method may identify patients overlooked by conventional screening who should undergo neuroimaging such as MRI. Based on our search of several databases, including PubMed, Embase, Scopus, and Google Scholar, no previous reports documenting this approach were found. Some of the methods used to screen for voice abnormalities in acute ischemic stroke include the Frenchay Aphasia Screening Test (FAST), the Aphasia Rapid Test, and the Language Screening Test (LAST). These tests evaluate the patient’s speech during conversation, assess their ability to produce phrases or words, and may involve tasks such as repeating phrases or words [10,11,12]. However, it should be noted that these methods do not provide a quantitative assessment of changes compared to the patient’s previous voice state, at least in the emergency department [10,11,12]. In this case, in addition to observing any abnormalities in the patient’s conversation, we conducted a speech assessment using a method commonly practiced in Japan. This method involves both producing and repeating explosive sounds, as well as repeating consonants, which is considered slightly more challenging. However, no abnormalities were found. Even the friend who was familiar with the patient’s voice did not notice any changes compared to the patient’s usual voice. This novel approach was developed by asking the patient to call out to his friend, who was positioned 5 m away, and evaluate his usual calling voice. Since the friend was well-acquainted with the patient’s typical speech characteristics, their perspective was leveraged to provide more precise insights into any changes observed, thereby improving the accuracy and comprehensiveness of neurological assessments. The presence of dysarthria was successfully detected using this method. If applicable, if the patient has established familiar phrases such as “make me some coffee” or “close the window” at distances of 5 m or 10 m, such phrases could also be used as part of the Calling Method. This further enhances the versatility and potential applications of this approach, expanding its scope. This approach has the potential to become a valuable tool for speech assessment in clinical settings, particularly for patients with neurological deficits like brainstem infarction. By enhancing the accuracy and comprehensiveness of neurological evaluations, healthcare professionals can improve treatment outcomes and patient care.

Traditionally, healthcare professionals have been solely responsible for detecting and diagnosing impairments in neurological assessments [1,14,15,16,17]. However, this report demonstrates the potential benefits of including the perspectives and observations of individuals close to the patient. This collaborative approach has the potential to detect weak dysarthria that may be missed in traditional neurological assessments.

This approach can also be applied to activities such as singing or playing musical instruments, as well as to patients who are unable to sit or stand independently. Additionally, in patients who are unable to sit or stand on their own, family members or friends can observe the patient’s movements, such as turning over or sitting up, and note any deviations from their usual behavior. In fact, it is important to have family members perform tasks in the examination room, such as assisting the patient in transitioning from lying down to sitting, to allow them to determine if there are any differences compared to the patient’s normal behavior.

The involvement of family members or friends in the assessment process represents a shift in healthcare, as it recognizes the importance of leveraging the collective knowledge and observations of those who are closely associated with the patient’s daily life.

Several potential areas of further research and exploration can be derived. Firstly, future studies could focus on conducting larger-scale investigations to validate the effectiveness and reliability of the Calling Method as a screening tool for detecting subtle voice changes associated with brainstem infarction. By including a more diverse patient population and multiple assessment sites, the generalizability of the approach can be better understood. Moreover, it would be valuable to compare the diagnostic accuracy of the Calling Method with existing neurological assessments and imaging techniques to determine its added value in clinical practice. Second, traditional neurological evaluations have been primarily conducted by professionals, while non-professionals such as family members or friends have played a supplementary role. It would be important to compare the ability of professionals and non-professionals to detect abnormalities in voice assessments. Additionally, the development of evaluation methods that are user-friendly for non-professionals would be necessary. Third, it is important to acknowledge the potential role of artificial intelligence (AI) in neurological assessments [18,19]. With advancements in machine learning techniques, AI has the ability to detect subtle changes in speech characteristics that may indicate neurological conditions like dysarthria. The utilization of large datasets of voice recordings, encompassing both everyday speech and voice during call-outs through the Calling Method described in this report, enables AI systems to potentially predict the expected normal “call-out” voice during patient examinations. By comparing it with the actual “call-out” voice of the patient, AI systems have the potential to surpass untrained human observers and clinicians in identifying these changes. However, it is crucial to emphasize that the development of an effective and reliable AI system necessitates careful consideration of ethical implications, including data privacy and informed consent. To ensure the accuracy and applicability of such systems in healthcare settings, rigorous testing and validation would be essential. The integration of AI in neurological assessments holds promise for enhancing diagnostic capabilities and improving patient care outcomes.

Limitations of this study include the reliance on a single case, which restricts the generalizability of the findings. The inclusion of a larger sample size is necessary to establish the reliability and effectiveness of the proposed approach. Additionally, potential biases associated with non-professional observers need to be addressed. To control for bias, standardized evaluation methods and training for observers could be implemented to ensure accurate and consistent observations. The relationship between the observer and the patient may also influence the observations, and further research is needed to understand how different relationships impact the assessment process. It is important to acknowledge that involving non-professional observers introduces ethical considerations, such as privacy, consent, and confidentiality, which should be carefully managed to protect the rights of the patient and maintain trust in the healthcare system.

## 5. Conclusions

Involving family and friends in neurological assessments is an unexplored area [1,14,15,16,20,21]. The proposed approach utilizes their insights to detect subtle speech changes, thereby improving diagnosis and patient care. This collaborative approach strengthens relationships and enhances the comprehensive evaluation of neurological function. This case report demonstrates the potential usefulness of the Calling Method as a screening tool to detect subtle voice changes associated with brainstem infarction, which may be missed by conventional neurological examinations. Further research is needed. The proposed method may be useful in identifying patients who require further neuroimaging, such as MRI.

## Data Availability

Not applicable.
